# RNA m^6^A modification: a key regulator in normal and malignant processes

**DOI:** 10.1016/j.clnves.2025.100023

**Published:** 2025-06-06

**Authors:** Lianjun Zhang, Yidan Lou, Weini Li, Hongshan Guo, Le Xuan Truong Nguyen, Zhenhua Chen

**Affiliations:** a Department of Hematological Malignancies Translational Science, Beckman Research Institute of City of Hope, Duarte, CA 91010, USA; b Zhejiang Provincial Key Laboratory of Hematopoietic Malignancy, Zhejiang University School of Medicine, Zhejiang University, Hangzhou 310058, China; c Department of Hematology, The First Affiliated Hospital, Zhejiang University School of Medicine, Zhejiang University, Hangzhou 310058, China; d Zhejiang University School of Medicine, Zhejiang University, Hangzhou 310058, China; e Department of Biomedical Sciences, Cedars-Sinai Medical Center, Los Angeles, CA 90067, USA; f Bone Marrow Transplantation Center of the First Affiliated Hospital & Liangzhu Laboratory, Zhejiang University School of Medicine, Hangzhou 310058, China; g Applied Cancer Research and Drug Discovery, Translational Genomics Research Institute, Phoenix, AZ 85004, USA

**Keywords:** RNA modification, M6A, CarRNA, Histone modification, Cancer, Eraser, Writer, Reader, Inhibitor, Mapping technology

## Abstract

The dedicated control of gene abundance is essential for both biological and pathological processes in mammals. Multiple layers of gene expression regulation, including transcriptional, post-transcriptional, translational, and post-translational regulation, collectively determine the highly dynamic equilibrium of functional protein abundance. Epigenetic modifications play indispensable roles in fine-tuning gene expression at either DNA, RNA, or protein level. To date, over 170 chemical modifications have been identified in RNA, with *N*^6^-methyladenosine (m^6^A) emerging as the most abundant and functionally significant modification in messenger RNA (mRNA). Many proteins have been identified as m^6^A-related proteins such as “writer” (deposition), “eraser” (removal) and “reader” (recognition). The dynamic m^6^A abundance (controlled by writer and eraser) together with reader proteins determine mRNA fate/metabolism, including transcription, alternative splicing, nuclear export, mRNA stability, and translation. Here, we summarize the latest findings on m^6^A-associated molecular mechanisms, emerging technologies for mapping m^6^A, and the roles of m^6^A-related proteins in both normal and malignant contexts. We further discuss/review the controversial opinions and open debates, and translational/clinical potential of m^6^A/m^6^A-related proteins as therapeutic targets, highlighting remaining questions and research directions in RNA m^6^A modifications.

## 1. Introduction

The dedicated equilibrium of gene abundance in mammals that orchestrates and regulates both biological and pathological processes is fundamental to maintaining cellular homeostasis. This regulation has multiple layers to determine the final abundance of functional proteins in a context-dependent manner, such as transcriptional, post-transcriptional, translational, and post-translational controls. Epigenetic modifications are the major factors that regulate gene expression from transcription to post-translation and thus globally fine-tunes the biological processes.

Among epigenetic modifications, RNA modifications play a crucial role in post-transcriptional gene regulation. To date, over 170 distinct chemical modifications have been identified in RNAs such as *N*^6^-methyladenosine (m^6^A), pseudouridine (ᴪ), *N*^6^, 2’-*O*-dimethyladeno-sine (m^6^Am), 5-methylcytidine (m^5^C), *N*^1^-methyladenosine (m^1^A), *N*^7^-methylguanosine (m^7^G) etc. [1] Among all the modifications, m^6^A (accounts for 0.4–0.7 % of adenosine in human mRNA) is the most abundant modification that determines mRNA fates including transcription, alternative splicing, and nuclear transport to cytoplasm, stabilization/degradation, translation initiation and elongation. [1–3] The deposition of m^6^A is catalyzed by a methyltransferase complex (“writers”), removed by demethylases (“erasers”), and recognized by specific RNA-binding proteins (“readers”), which collectively modulate gene expression. The dynamic and reversible nature of m^6^A has positioned it as a key regulator in diverse biological processes, including cell differentiation, immune responses, stress adaptation, and tumorigenesis.

More than 90 % of m^6^A sites conform to the canonical DRAC motif (D=G/A/T, R=A/G) by a novel single-base m^6^A methylation mapping approach and are randomly distributed across the mRNA transcripts but most enriched near stop codons and internal exons of nucleotides larger than 200 nt. [4,5] Exon junction complexes (EJCs) help prevent depositions of m^6^A modifications into coding region and therefore shows a preference of binding near stop codons. [6] Additionally, RNA binding proteins (RBPs) which interact and recruit m^6^A deposit/removal/reorganization machinery also modulate the mRNA fates in both normal and abnormal contexts. [7].

Despite significant progress in understanding the fundamental biology of m^6^A, several questions remain unanswered. How m^6^A deposition is precisely regulated in a context-specific manner, how different RNA modifications cross-talk to influence gene expression, and how aberrant m^6^A modifications contribute to disease pathogenesis are still open questions in the field. Additionally, technological advancements are needed to improve the resolution and specificity of m^6^A mapping approaches.

In this review, we summarize the latest discoveries on the molecular mechanisms and open debates of m^6^A modifications, as well as novel technologies to map the m^6^A-epitranscriptome, and the roles of m^6^A-related proteins in both normal and malignant contexts. Furthermore, we discuss the translational and clinical potentials of m^6^A/m^6^A-related proteins as either biomarkers for diagnosis or therapeutic targets for clinical treatment. Collectively, we provide a comprehensive overview of m^6^A modification, highlighting the emerging roles, uncertain aspects and future directions in determining mRNA fates.

## 2. Proteins of m^6^A machinery

**Fig. 1.**
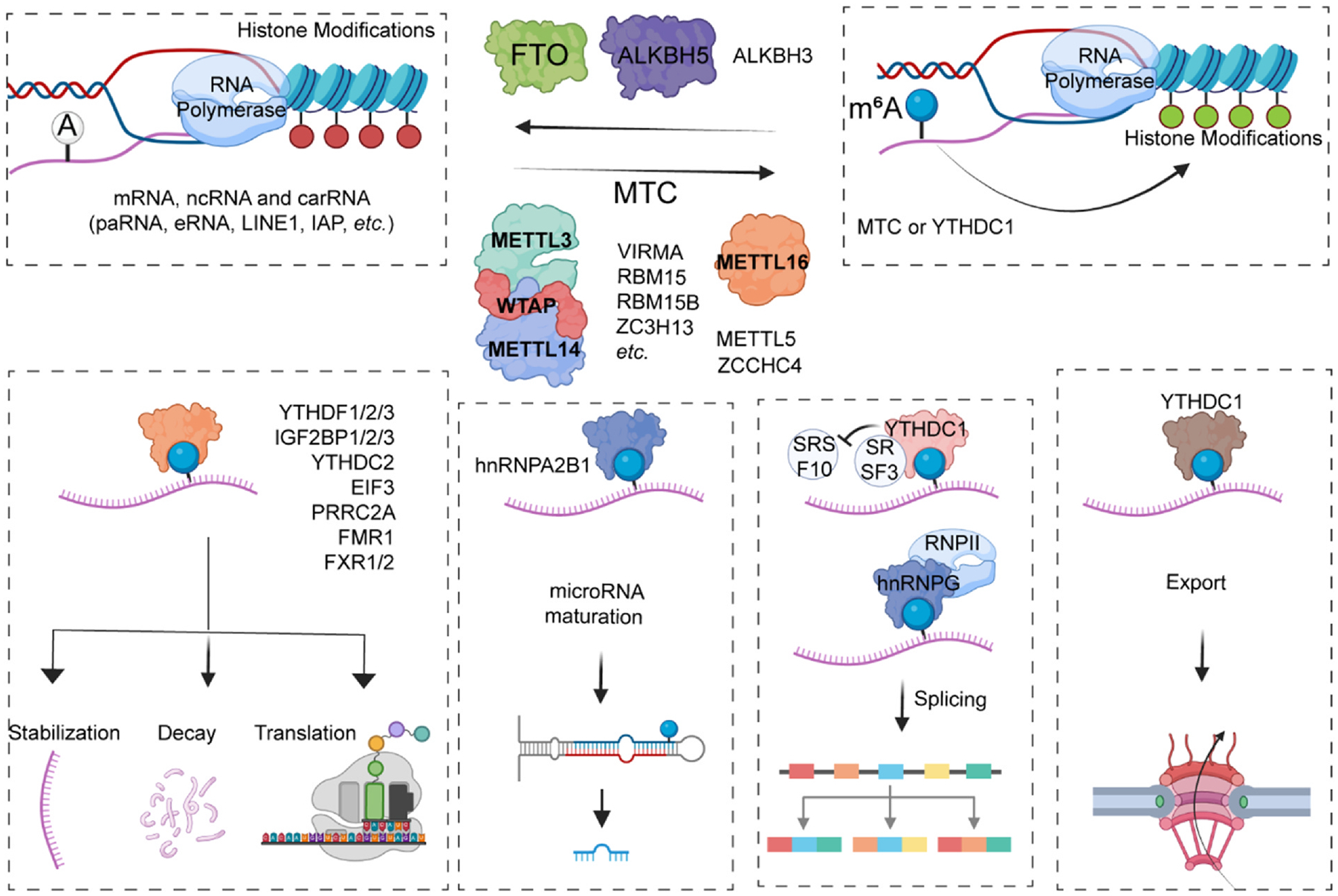
RNA m^6^A machinery (writer, eraser, reader) in regulating gene expression by cross-talking with histone modifications, and mRNA fates such as mRNA stability, decay, translation, splicing, microRNA processing and exportation.

Similar to histone methylations which were crucial and highly dynamic epigenetic processes evidenced by the discoveries of the methyltransferases (chromatin writers) and demethylases (erasers) in the past decades, [8] the critical proteins involved in RNA m^6^A modification machineries are also defined as m^6^A writers (deposit m^6^A), erasers (remove m^6^A) and readers (recognize m^6^A) (Fig. 1). [3] The major methyltransferase complex consists of two methyltransferases (METTL3-METTL14) and other scaffold proteins such as WTAP, VIRMA, RBM15-RBM15B, ZC3H13, which enhance substrate recruitment and recognition of specific RNA-binding sites in nuclear speckles. [9–12] MTC such as METTL3, METTL14 and FTO cross-talk with histone modifications and regulate gene expression by interacting with m^6^A modification in chromosome-associated regulatory RNAs (carRNAs) (Fig. 1). [12,13] METTL3 depletion results in a reduction of m^6^A methylation, elevates the levels of carRNAs and promotes open chromatin state and downstream transcription. [13] Whether other writer proteins are involved in this process or exhibit similar functions in nucleus remains uncovered. Additionally, METTL16, METTL5 and ZCCHC4 have also been found working as methyltransferases and their substrates expand to long non-coding RNA, small nuclear RNAs and ribosomal RNAs. [14–16].

Compared to writer proteins and other scaffold proteins involved in installing m^6^A marks, only two dioxygenases (FTO and ALKBH5) have been reported and play crucial roles in removing m^6^A modifications, indicating that m^6^A signal is also a highly dynamic process. [17,18] Besides removing m^6^A marks in mRNA, FTO also plays a role in removing m^6^Am in mRNA and m^1^A marks in transfer RNAs. [19–22] Thus, m^6^A eraser plays a crucial role in chromatin state and gene expression by modulating the carRNAs in mammals. [13,22] ALKBH5 is another eraser enzyme negatively regulating m^6^A amounts in mRNA, [17,18] but whether it possesses the similar activity of modulating m^6^Am or m^1^A as FTO remains elusive. Interestingly, other interacting proteins involved in assisting the process of removing m^6^A marks have not yet been investigated. Recently, ALKBH3 was also reported to be capable of removing m^6^A modification in tRNA. [23] There is still an open debate about whether the m^6^A modification is reversible or not. [24] It still requires more studies from different research groups to verify that m^6^A removal is for sure dependent on FTO or ALKBH5/3.

Different RNA binding proteins have been found to recognize m^6^A modification such as YT521-B homology (YTH) domain containing family proteins (YTHDF1/2/3 and YTHDC1/2), insulin-like growth factor 2 mRNA-binding proteins (IGF2BP1/2/3), nuclear ribonucleoproteins (hnRNPA2B1, hnRNPC and hnRNPG), eukaryotic initiation factor 3 (EIF3), PRRC2A and fragile X messenger ribonucleoprotein 1 (FMR1) and related paralogs FXR1/2. [10,25–35] Moreover, Ras GTPase-activating protein-binding protein 1 (G3BP1) and G3BP2 have been reported to serve as m^6^A anti-readers which will be repelled by m^6^A modifications. [30,31] YTHDF2 induces mRNA decay by transferring m^6^A-modified mRNA to P-body, a degradation place in cytoplasm, and destabilizing mRNAs by recruiting CCR4-NOT deadenylase complex. [36] It has also been shown that YTHDF2 degrades/cleavages m^6^A-modified mRNAs by recruiting RNaseP/MRP complex. [37] The most recent study in cancer contexts shows that YTHDF2 promotes mRNA stability as an m^5^C reader protein by recruiting PABPC1. [38] YTHDF1 has been shown to facilitate ribosome loading and mRNA translation. [26] YTHDF3 enhances YTHDF1 in promoting translation efficiency and YTHDF2-mediated mRNA degradation. [39] The functional distinctions of YTHDF1, YTHDF2, and YTHDF3 remain debated, as they share a highly similar RNA binding pocket YTH domain. Several studies reported that that YTHDF family proteins usually function together to mediate the degradation of m^6^A-modifed mRNAs, suggesting a redundant function in regulating m^6^A-modified mRNA fates. [40] Intriguingly, a follow-up report showed that the different roles of YTHDF proteins are determined by the post-translational modifications and phase separation of N terminal. [41–43] Notably, a recent study suggests that the key amino acids of RNA binding pockets may contribute to the function and specificity of YTHDF2 with YTHDF1/3. [38] However, more evidence and mechanistic studies are still required to conclude.

YTHDC1 localizes in nucleus and regulates selective splicing of precursor RNAs by recruiting splicing factor serine and arginine-rich splicing factor 3 (SRSF3) while blocking the binding of SRSF10. [28,44] Moreover, YTHDC1 co-transcriptionally interacts with histone demethylase such as KDM3B and further promotes gene expression in an m^6^A-dependent manner in nucleus (Fig. 1). [45] YTHDC2 is the largest member of YTH family protein and binds to m^6^A-modified mRNAs to further promote translation efficiency or reduce their stabilities. [27].

IGF2BP family proteins promote m^6^A-modified mRNA stability by interacting with mRNA stabilizers such as ELAVL1, MATR3 and PABPC1, and meanwhile enhance mRNA translation by recruiting eukaryotic translation initiation factor proteins. [29] The four common RNA-binding K homology (KH) domains in IGF2BP proteins were responsible and required for selective binding with m^6^A modified RNAs, among which the KH3–4 di-domain is indispensable for m^6^A reorganization whose mutation (GXXG to GEEG) resulting in completely abolish of binding capacity with m^6^A. [29] Given the binding affinity of IGF2BP proteins with regular RNAs, the precise mechanism underlying the m^6^A selectivity and even whether they exhibit preference with other types of methylated RNAs require more investigation. Interestingly, a most recent study showed that IGF2BP family proteins IGF2BP1–3 can preferentially bind internal mRNA m^7^G. [46] A recent report showed that IGF2BP proteins stabilize m^6^A-modified target genes and protect them against miRNA-directed decay, indicating another miRNA-dependent mechanism of IGF2BP family proteins. [47].

HnRNPA2B1 is found to recognize m^6^A modification in primary microRNAs (pri-miRNAs) and promotes the processing and maturation of METTL3- “writing” newly transcribed pri-miRNAs by recruiting canonical miRNA maturation processors such as DROSHA and DGCR8. [33,48] Increasing evidence showed that m^6^A marks in mRNA, long non-coding RNA (lncRNA), and pri-miRNAs (pri-miR-21) control the RNA-structure-dependent accessibility of RBPs such as hnRNPC to affect RNA-protein interactions for biological regulation, which mechanism is termed “m^6^A-switch”. [32] HnRNPG is another m^6^A reader protein (via RRM and Arg-Gly-Gly (RGG) motifs) which can directly bind to the phosphorylated carboxy-terminal domain (CTD) of RNA polymerase II suing RGG motifs in its low-complexity region and further regulate alternative splicing transcriptome-wide co-transcriptionally in an m^6^A-dependent manner. [31,32] EIF3 directly binds to the 5′-UTR of m^6^A-modified mRNA and initiates mRNA translation. [34] Interestingly, this point is also controversial without conclusion so far, based on several follow-up studies showing that m^6^A in 5’-UTR does not promote translation initiation, which may be an indirect process regulated by YTHDF1 or YTHDF3. [26,49] More advanced and faithful methods are required to obtain new evidence to make a conclusion. PRRC2A is a newly identified m^6^A reader protein, which co-localizes but competes with YTHDF2 for m^6^A-modified RNA binding, and oppositely stabilizes mRNA stability. [35] RBPs containing KH domains and RGG motifs such as FMR1 and FXR1/2 are also reported as m^6^A reader proteins which facilitate target genes transportation from nucleus into cytoplasm. [30] Thus far, extensive research has identified a set of family proteins as m^6^A readers based on the current methods (RNA pull-down and LC-MS/MS, etc.), which may also expand in the future. An open question is whether any RBP containing RRM, KH, or RGG domains/motifs could bind to m^6^A-modified RNAs and even other types of modifications in different contexts, thus, the affinity and specificity of the m^6^A reader proteins should be redefined.

## 3. Technologies for m^6^A profiling and modifiers identification

Although the m^6^A stoichiometry is dynamically controlled by multiple modifiers and further determined by other layers of regulation such as intrinsic and extrinsic factors, diverse abundance of m^6^A “switch”/ reader proteins or TFs/RBPs which interact with m^6^A-modifiers, globally mapping transcriptomic m^6^A remains critical for the confirmation of the regions/sites of m^6^A marks in any specific mRNAs and the diagnosis/prognosis of multiple diseases serving as a bio-marker. Therefore, sensitive and reliable methodologies with high resolutions (single base) for m^6^A profiling are increasingly and urgently needed to be developed for further exploration of transcriptomic m^6^A marks. Several high throughput techniques specifically for m^6^A profiling have been developed and reported.

### 3.1. Antibody-based m^6^A-seq

**Fig. 2.**
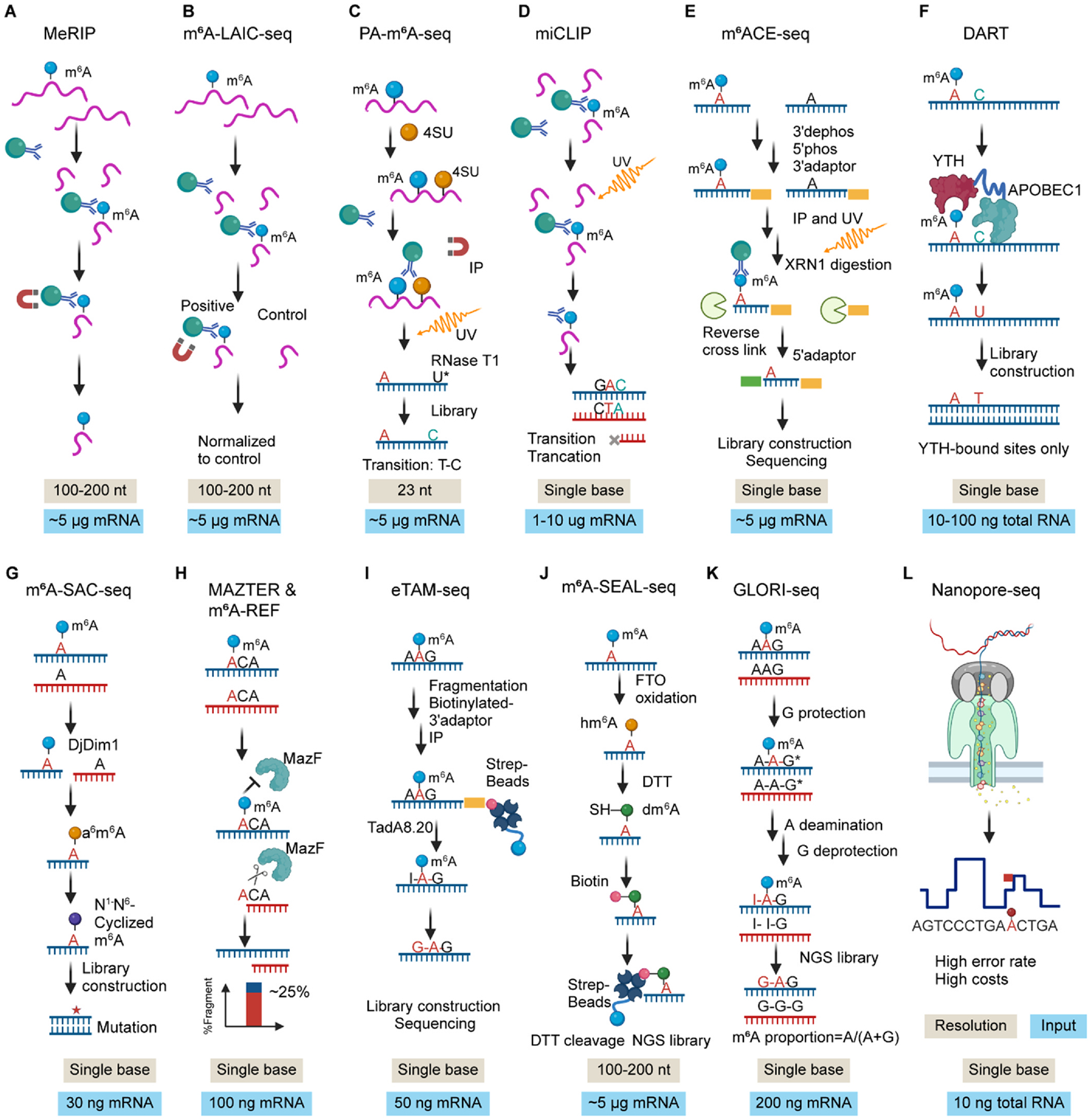
Reported technologies for mapping m^6^A signals in transcriptome. (A-E) Antibody-based methods. (F-K) Non-antibody-based methods. (L) The third-generation of sequencing technology: nanopore sequencing.

Methylated RNA immunoprecipitation followed by RNA sequencing (MeRIP-seq) is the first reported and most widely used antibody-based method for m^6^A profiling so far. MeRIP-seq requires a minimum of 5 μg mRNA or 30 μg total RNA as the starting material and provides the information on transcriptomic m^6^A modified regions in mRNA at a resolution of around 100–200 nucleotides (Fig. 2A). [5,50] This original method has two limitations: it requires a large amount of RNA and its resolution is not high enough. To overcome these limitations, several improved antibody-based methods have been developed such as PA-m^6^A-seq (resolution: ~23 nt, rely on 4SU), [51] miCLIP/m^6^A-CLIP (single-base but low reproducibility), [52,53] m^6^ACE-seq (single-base, but absolute percentage quantification is not available), [54] m^6^A-LAIC-seq (normalized control samples are included), [55] and SLIM-seq (super low input as low as 100 ng for total RNA and 10 ng for synthesized transcripts)^56^ (Fig. 2B–2E). Though the single base resolution could be reached by antibody-based method, current antibody strategy could hardly discriminate m^6^A modification from DNA *N*^6^-methyladenine (6 mA) nor m^6^Am, in line with observed biases towards specific RNA sequences or secondary structures. Overlapping with RNAs bound to m^6^A-related protein and systematic calibration using a synthetic modification-free RNA library are required to increase the accuracy and selectivity of antibody methods. Moreover, quantitative reverse transcription PCR (qRT-PCR) by Bst I enzyme for specific modified A residue is also an ideal option for verification of specific sites. [57].

### 3.2. Antibody-independent m^6^A-seq

To reach a durable single-base resolution using a small amount of RNA, several antibody-independent m^6^A-seq have been developed such as MAZTER-seq (single base, only ~25 % m^6^A sites can be detected), [58] m^6^A-REF-seq (single base, only ~25 % m^6^A sites can be detected), [59] m^6^A-SEAL (200 nt, 5 μg mRNA), [60] DART-seq (single base, as low as 10 ng total RNA, only rough quantitate m^6^A levels bound to YTH domains), [61] m^6^A-SAC-seq (single base, 30 ng mRNA, less sensitive to A (m^6^A) C motif, cover ~65 % of transcripts identified by antibody-based method, rely on recombinant DjDim1 which is not commercially available so far), [62] GLORI (single base, 200 ng mRNA, unable to tell m^6^A from m^6^Am/m^1^A) [63] and eTAM-seq (single base, 50 ng mRNA, less sensitive to low abundance m^6^A, unable to tell m^6^A from m^6^Am/m^1^A) (Fig. 2F–2K). [64] These methods somehow largely expand the current strategies mapping m^6^A-seq at a single-base resolution using super low input. More advanced and applicable methods are required for future potential biomarker research and applications for some disease diagnoses and even mapping the real-time m^6^A in a single cell & single-base resolutions.

### 3.3. Third-generation sequencing: nanopore sequencing

All the RNA-seq data based on cDNA method which uses reverse transcription might introduce biases and have some limitations. The third-generation sequencing method employing Oxford Nanopore technology could circumvent these limitations through direct sequencing of native RNA. [65] When RNA passes through the pore, magnitudes of electric intensity across the nanopore surface are recorded and further used to confirm the corresponding nucleotide information. RNA modifications cause shifts in intensity levels that could be used to identify modified bases after bioinformatic analysis (Fig. 2L). However, systematic evaluations reveal trade-offs between precision and recall across computational tools, with most limited to detecting m^6^A in DRACH motifs (e.g., GGACT/AGACT) and showing poor sensitivity in non-DRACH contexts due to subtle current variations.

The third-generation RNA-seq has been used to analyze m^6^A in yeast, Arabidopsis, RNA virus genomes and human cells. [66–69] Recently, an updated computational method, xPore, was developed to identify positions of m^6^A sites at single-base resolution using the third generation nanopore sequencing data. [65] Compared to existing approaches such as m^6^ACE-seq, miCLIP or m^6^A-MAZTER-seq, nanopore RNA-seq not only provides a higher precision more efficiently, but also identifies more accurate positions containing DRACH motifs. Notably, xPore achieves superior quantitative accuracy but requires high sequencing coverage (≥5 reads/site) and near-complete m^6^A depletion in controls (e.g., *Mettl3* KO), limiting its clinical utility where ideal controls are unavailable. Nanopore sequencing followed by xPore analysis achieves base resolution for m^6^A and enables quantitative comparison of samples in the absence of a control sample by estimating the modification rate. Therefore, xPore allows jointly modeling RNA modification rates across a large amount of data and unmatched loss-of-m^6^A control, which is essential for patient samples and primary tissues.

In addition, a living cell-based technology called GEMS (genetically encoded m^6^A sensor) has been developed for detecting changes in m^6^A according to the readout of GFP fluorescent signal, which may help screen the pharmacological inhibitors targeting m^6^A levels in cells (Table 1). [70] The accurate and durable technology may advance future drug development in tumors targeting m^6^A levels or related proteins.

### 3.4. Identifying m^6^A binding proteins

RNA pull-down followed by LC-MS/MS is the major method which is currently used for the screening and verification of proteins that recognize and bind to m^6^A marked RNAs. [71] To further confirm the binding includes MicroScale Thermophoresis (MST) assays to measure the Kd value, confocal analysis of m^6^A-modified RNA with the locations of proteins by fluorescence in situ hybridization (FISH) and immunofluorescence assay in cells and global overlapping analysis of m^6^A marks with protein-bound sites, etc.

The current methods have advanced our understanding of m^6^A distribution and profiling in either normal or tumoral contexts. Although the antibody-based or independent methods have advantages or some limitations, the m^6^A signals detected by different methods are consistent. Newly developed single-nucleotide quantitative methods require special recombinant enzymes, show a moderate coverage of m^6^A-modified transcripts or limited capacity for distinguishing m^6^A with m^6^Am/m^1^A. In contrast, the antibody-based methods (m^6^A-RIP) together with some qPCR detections such as Bst PCR, which are more accessible and popular, usually show the biggest coverage and single-nucleotide resolution. New methods with better coverage and high specificity to m^6^A (not m^6^Am or m^1^A) at a single-nucleotide and single-cell resolution are warranted. In addition, more methods to detect low cell populations such as stem cells or extracellular exomes are also required.

## 4 m^6^A modification in normal cellular processes

4.

### 4.1. Cross-talk of RNA m^6^A and histone modification

**Fig. 3.**
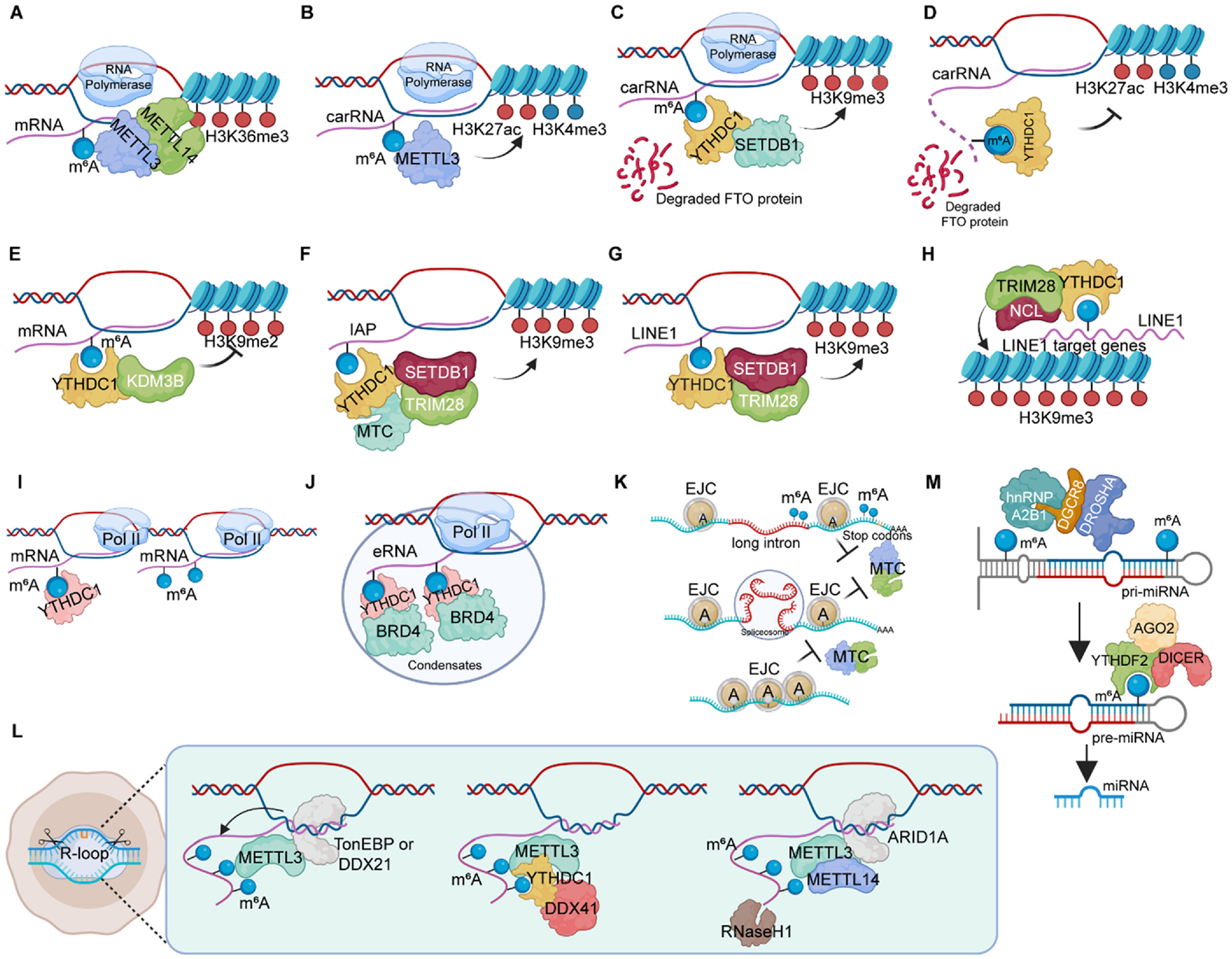
Reported molecular mechanisms that m^6^A writer or reader proteins regulate histone modifications and microRNA processing. (A) H3K36me3-METTL14-RNA Pol II axis. (B) METTL3-mediated carRNA methylation. (C-D) FTO-carRNA-m^6^A-YTHDC1 regulates histone modifications and gene expression. (E) YTHDC1-m^6^A-mRNA cross-talks with H3K9me2 by interacting with KDM3B. (F-H) YTHDC1-TRIM28/SETDB1 axis in LINE1 regulation. (I) YTHDC1-m^6^A-mediated RNA Pol II pause release. (J) LLPS formation by m^6^A-YTHDC1-BRD4. (K) EJC-mediated the distribution of m^6^A modifications in mRNA. (L) RNA m^6^A system in R-loop and genome stability maintenance. (M) RNA m^6^A-mediated miRNA maturation.

H3K36me3 is the first reported histone modification that cross-talks with RNA m^6^A modification. Mechanistically, m^6^A MTC member METTL14 recognizes H3K36me3 and recruits RNA Pol II to deposit m^6^A on newly synthesized RNA (Fig. 3A). [12] It has been reported that METTL3 could deposit m^6^A modifications on chromosome-associated regulatory RNAs (carRNAs), including promoter-associated RNAs, enhancer RNAs and repeats RNAs (Fig. 3B). [13] Similar to writer proteins, FTO promotes open chromatin state and gene expression by mediating the m^6^A modification abundance in long-interspersed element-1 (LINE1) RNA, which in turn modulates the transcription of LINE1-containing genes (Fig. 3C–3D). [22] Similarly, YTHDC1, a reader protein located in nucleus, promotes m^6^A-modified genes transcription by interacting with H3K9me2 demethylase KDM3B and further open the chromatin state (Fig. 3E). [45] Then, another study shows that *METT14* promoter is enriched with H3K4me3, which is mediated and removed by KDM5C. [72] *ALKBH5* promoter is also enriched by H3K9me3 demethylase KDM4C, which will further promote MYB and Pol II expression in leukemia cells. [73] EZH2 binds to and promotes METTL3 expression by maintaining the H3K27ac modification abundance at METTL3 promoter. Reversely, m^6^A modification on *EZH2* mRNA facilitates genomic H3K27me3 abundance. [74] In bacterial infection, *YTHDF2* KO stabilizes m^6^A-modified *KDM6B*, coding a H3K27me3 demethylase, and promotes the demethylation of H3K27me3 and transcription of target genes. [75].

### 4.2. Chromosome associated regulatory RNAs and chromatin state

Recently, chromosome associated regulatory RNAs (carRNAs), as an emerging subset of nuclear RNAs that control the chromatin state, have been found play critical roles in chromatin accessibility and transcription activation. Reducing m^6^A methylation by *METTL3* KO or site-specific m^6^A demethylation of selected carRNAs enhanced the levels of carRNAs such as promoter-associated RNAs (paRNAs), enhancer RNAs (eRNAs) and transposable elements (repeats RNAs) and further promotes open chromatin state and downstream transcription. [13,45,76–78] It is reported that METTL3/14 KO increased the abundance of intracisternal A-particles (IAPs), a kind of long terminal repeat elements (LTRs), without changing chromatin modifications such as H3K4me3, H3K27ac and H3K9me3. [79] In contrast, another study reported that *Mettl3* KO significantly reduced repressive histone marks such as H3K9me3 and H4K29me3 by regulating the IAP genomic loci but not elevating IAP expression in mESCs (Fig. 3F). [78] YTHDC1’s recognition of m^6^A marks in IAP and simultaneously recruiting of TRIM28 and SETDB1 is a major mechanism of inhibiting an open state of chromatin via H3K9me3 (Fig. 3F).^76^It has also been reported that m^6^A-modified LINE1 represses gene transcription via a mechanism of YTHDC1-m^6^A-TRIM28-SETDB1 complex, leading to a close chromatin regulated by H3K9me3 (Fig. 3G). [77] Additionally, another group also reported that YTHDC1 and m^6^A-modified LINE1 form a scaffold to induce the TRIM28-NCL complex to deposit the H3K9me3 to LINE target genes loci (Fig. 3H). [80] The m^6^A MTC and YTHDC1 are recruited to gene promoters and depleting the m^6^A leads to a decrease in RNAP II pause release (Fig. 3I). [81] Interestingly, *METTL16* KO resulted in a greater reduction of YTHDC1 binding to LINE1 than *METTL3* KO in mESCs, [77] whether METTL16 is more responsible for m^6^A abundance in LINE1 and its critical roles in chromatin and transcription remains unclear. All the studies were conducted in mESCs in the papers mentioned above, whether this mechanism works in more differentiated cells or other species remains elusive. H3K9me3 modification over LINE1 genomic loci is not only regulated by SETDB1 but also SUV39H1/2 (another H3K9me3 methyltransferase). The dynamic and dedicated regulation of LINE1 loci via m^6^A mechanism requires more investigation. It is controversial that LINE1 expression level is opposite after *METTL3* KO in the same tissues. There is also an open question about how YTHDC1-SETDB1-TRIM28-H3K9me3 selects or distinguishes the different types of m^6^A-modified RNAs such as eRNAs, paRNAs, LINE1, etc.

Moreover, it has been reported recently that *FTO* KO promotes the close chromatin state and gene inactivation, via YTHDC1-LINE1 axis during mouse oocyte and embryonic development (Fig. 3C). [22] Consistently, YTHDC1 forms a complex with RBFOX2, a newly identified m^6^A-caRNA reader protein, and suppresses transcription and chromatin state by recruiting PRC2 protein complex. [82] On the contrary, another report also demonstrated that YTHDC1 binds to m^6^A-modified eRNAs and further forms the coactivator condensate with BRD4 to activate gene expression in multiple cancer types of cells such as leukemia (K562), breast cancer (MCF-7) and cervical cancer (Hela) (Fig. 3J). [83] Therefore, whether the activation or inactivation of YTHDC1-m^6^A-eRNA on gene transcription relies on different cell contexts (cancers vs mESCs) remains an open question.

A recent report showed that EJCs suppress m^6^A in coding region by preventing MTC’s accessibility and globally shape the m^6^A specificity on 3’ UTR regions (Fig. 3K). [6] In addition to m^6^A, chromatin-associated retrotransposon RNA m^5^C, which can be oxidized by TET2 and antagonized its MDB6-dependent H2AK119ub deubiquitination, promotes an open chromatin state and plays key roles in leukemogenesis. [84] Whether other modifications of carRNAs in nucleus also play similar roles as m^6^A remains elusive. The cross talk of m^6^A-histone modification opens a new revenue of RNA modifications with protein modification involving the process of chromatin state and gene transcription.

### 4.3. R-loop

R-loops, a type of three-stranded nucleic acid structure which was observed in 1976, consist of an RNA strand that pairs with one of the template strands of double-stranded DNA leaving the other DNA strand free. [85] R-loop formation usually leaves the other unpaired DNA free and widely present in the mammalian genome (about 5 %) at the promoter and transcriptional termination regions. [86] Physiological R-loops play crucial and broad roles in various biological processes including cell proliferation/differentiation, DNA repair, DNA methylation and gene regulation (e.g. transcription, replication, recombination, centromere function and DNA editing), despite its original harmful roles as by-products in transcription and genomic instability. [87,88] Pathological R-loop formation which is caused by poorly management and control usually results in aberrant gene expression and diseases such as cancers. [86] Recent years, m^6^A modification has also been found enriched in R-loop and plays a role in maintaining genome stability. [89] Moreover, another short report found that m^6^A promotes R-loop formation and facilitates transcription termination. Mechanically, TonEBP recognizes R-loops and initiates m^6^A modification by recruiting METTL3, and the deletion of TonEBP or METTL3 will increase R-loop formation and decrease cell survival (Fig. 3L, left). [90] However, a most recent report found that DDX21 can also recognize R-loop and promote transcription termination maintaining genome stability by recruiting METTL3. The deletion of loss of enzymatic function of these two proteins will lead to DNA damage (Fig. 3L, left). [91] Moreover, YTHDC1 recognizes m^6^A-modified R-loops and participates in DNA damage responses induced by R-loops (Fig. 3L, middle). [92] ARID1A is another critical protein which recruits METTL3/14 to R-loops and promotes HR repair in response to DNA damage (Fig. 3L, right). [93] m^6^A modification in R-loops facilitates genome stability during DNA damage repair by recruiting RNaseH1. [93] Although the consequences (e.g., cell survival, DNA damage and genome stability) of m^6^A-deficiency by deleting writer proteins are consistent in many studies, the role of RNA m^6^A modification in R-loops formation remains to be further clarified by studying more m^6^A related proteins such as eraser and reader proteins.

### 4.4. miRNA processing

Both m^6^A modification and microRNAs (miRNAs) are key post-transcriptional regulatory mechanisms that control gene expression by modulating mRNA stability and translation. They also share the same 3′-untranslated regions (UTRs) regions for their target gene, which suggests potential cross-talk between these two regulators. Recent studies have revealed intricate interactions between m^6^A modifications and miRNA biogenesis and function, highlighting a complex regulatory network. [94].

MiRNAs are a class of small noncoding RNAs (~21 nt) and mature microRNAs are generated from primary transcripts (pri-miRNA) by microprocessor DGCR8 and DROSHA and further processed by DICER into precursor microRNA (pre-miRNA). Several studies have shown that METTL3-deposited m^6^A modification exists in pri-miRNAs and enhances the recognition and binding of DGCR8 to the substrate, promoting microRNA maturation, which is also mediated and recognized by HNRNPA2B1 (Fig. 3M). [32,33] Depletion of METTL3 led to reduced DGCR8 binding, resulting in fewer mature miRNAs and accumulation of unprocessed pri-miRNAs. In addition to interaction with DGCR8, METTL3 has also been shown to facilitate the synthesis of mature miRNAs by promoting the splicing of pre-miRNAs by Dicer. [95] For example, one study reveals that METTL3-mediated m^6^A methylation enhances miR-335 maturation, which in turn promotes stress granule (SG) formation and reduces apoptosis in ischemic neurons. ^96^In summary, these findings demonstrate that METTL3 plays an essential role in promoting the occurrence and development of tumors by enhancing the synthesis of tumor-promoting factor miRNAs in various cancers.

Similarly, METTL14 also plays a role in cancer as METTL3, which also promotes the recognition and binding of DGCR8 to m^6^A-modified pri-miRNAs, thus facilitating miRNA maturation. Studies have shown that METTL14 promotes pri-miR-126 maturation through the recruitment of DGCR8-dependent m^6^A in hepatocellular carcinoma (HCC). Deletion of METTL14 in HCC cells reduces m^6^A levels and miR-126 expression, resulting in enhanced migration and invasion of cancer cells. [97] Additionally, METTL14 was observed abnormal expression in breast cancer tissues and cells, and overexpression of METTL14 promoted the migratory and invasive abilities of breast cancer cells by increasing miR-146a-5p expression. [98] Moreover, m^6^A modification is also observed in pre-microRNAs (e.g. pre-miR-126) and promotes the microRNA maturation via YTHDF2. [99] However, whether YTHDF2 regulates the maturation of a set of microRNAs in both normal and abnormal contexts remains elusive.

MiRNAs play crucial roles in inhibiting gene expression by complementary binding to target mRNA 3’UTR region in RNA-induced silencing complex (RISC). AGO2 (Argonaute 2) is a key protein in the RNA-induced silencing complex (RISC) that plays a central role in the function of microRNAs (miRNAs). One study uncovers a regulatory mechanism in which m^6^A modification enhances miR-133a-mediated repression via the AGO2-IGF2BP2 complex during heart development and hypertrophy. IGF2BP2, physically interacts with AGO2, increasing miR-133a accumulation at m^6^A-modified target sites. [96] Overall, emerging studies highlight the interactions and regulatory roles between m^6^A modification and miRNAs, offering valuable insights for future research.

### 4.5. m^6^A in stem cell fate and cell reprogramming

Epigenetic regulations such as DNA methylation and histone modification have been reported to play critical roles in early embryonic development, self-renewal, and differentiation of stem cells. As the most abundant mRNA modifications, m^6^A is also reported to play indispensable roles in regulating stem cell fates. It has shown that m^6^A methylomes in human and mouse embryonic stem cells (ESCs) are highly conserved and its loss promotes ESC self-renewal and hinders differentiation. [100] In contrast, another study showed that deletion of Mettl3 or Mettl14 leads to m^6^A loss and lost capacity of self-renewal in mouse ESCs (mESCs) via human antigen R (HuR)-microRNA regulated degradation pathways. [101] Moreover, METTL3 knockout (KO) impairs self-renewal and triggers differentiation of porcine induced pluripotent stem cells (iPSCs) through YTHDF1/3-regulated JAK2-STAT3 pathway. [102] *Zfp217* depletion compromises ESC self-renewal and somatic cell reprogramming by releasing Mettl3 thus increasing m^6^A modification abundance and further enhancing degradation of m^6^A-mediated *Nanog*, *Sox2*, *Klf4*, and c-*Myc* mRNAs. [103] *METTL3* deletion or m^6^A loss prolonged *NANOG* expression, thus promoting mESCs self-renewal and blocking differentiation. [104] *Mettl3* KO inhibited the mouse primed epiblast stem cell (EpiSC) reprogramming efficiency to naïve pluripotency in early stages, which will be opposite in late stages. [104] In contrast, METTL3 overexpression results in the increased colony numbers of iPSCs and pluripotent factors expression in an m^6^A-dependent manner. [105] *Zc3h13* facilitates m^6^A methylation and promotes mESCs self-renewal by anchoring WTAP, Virilizer, and Hakai in the nucleus. [106] Notably, in human ESCs, SMAD2/3 has been shown to promote m^6^A deposition onto nascent transcripts by interacting with m^6^A MTC and exit from pluripotency toward lineage-specific differentiation. [107].

In a zebrafish model, m^6^A reduction by *Mettl3* KO blocks endothelial-to-hematopoietic transition (EHT) and decreases the hematopoietic stem/progenitor cell (HSPC) population by protecting Notch1a from YTHDF2-mediated decay. [108] Another subsequent study showed that *Mettl3* KO inhibits proliferation and promotes HSPC differentiation by inhibiting C-MYC, BCL-2 and PTEN translation and stimulating ATK signaling pathway. [109] Mettl14 is highly expressed in HSPCs, and its KD promotes terminal myeloid differentiation by suppressing MYB/MYC. [110] On the contrary, other groups showed that *Mettl3* and/or *Mettl14* KO impairs HSCs fates (e.g., symmetric, commitment, identity, self-renewal, and differentiation) and thus leads to HSCs accumulation and expansion. [111] Based on these studies, the consistency of m^6^A in determining HSCs or HSPCs fates is not good, therefore further investigation of the m^6^A-independent roles of the writer proteins in HSCs is required to understand the observations. Moreover, several groups reported that *Mettl3* or *Mettl14* KO in germ cell-specific lineages results in inhibition of spermatogonia stem cells (SSC) proliferation/differentiation by regulating target mRNA translation. [112,113] Interestingly, *ALKBH5* KO also showed a deficiency in spermatogenesis or male fertility in mice. [18] Although different target genes were identified, the underlying mechanisms of either writer or eraser protein deletion could result in a similar impaired phenotype of spermatogenesis warrant further studies. Given the current hypothesis that m^6^A is dynamic, which means the target genes of writer proteins should also be the targets of eraser proteins, the mechanisms of m^6^A in regulating spermatogenesis remain unclear, whether these proteins play roles in an m^6^A-independent mechanism is unknown. Moreover, *Mettl3* KO in mesenchymal stem cells inhibits osteogenic differentiation by regulating the translation efficiency of Pth1r in an m^6^A-dependent manner. [114–116] Other underlying mechanisms or target genes have also been reported and identified such as PI3K-AKT, VEGFa, ATP6V0D2, TRAF6, MAPK, NF-ĸB, etc. [115,116] In addition, *YTHDF2* KO promotes HSC expansion but inhibits leukemic stem/initiation cells (LSCs/LICs) via m^6^A-depnedent pathways. [99,117] *Ythdc1* and *Ythdc2* KO result in impaired spermatogenesis and male fertility in mice. [18,118].

During preimplantation embryo development, the global RNA m^6^A abundance decreased from the stage of germinal vesicle to the two-cell stage and increased after then. *METTL3* KO inhibits transcription in the two-cell stage by regulating translation efficiency and mRNA degradation. [119] YTHDF2 sustains oocyte maturation and early embryonic development by mediating maternal mRNA degradation in an m^6^A-dependent manner. [120,121] IGF2BP1 KO results in impaired early parthenogenetic embryogenesis. [122] Intriguingly, IGF2BP2 is highly expressed in oocytes and IGF2BP2-depleted mice not only show female infertility but also embryos blocked at two-cell stage by suppressing the transcription and translation of target genes via m^6^A modification. [123] In a study of zebrafish, IGF2BP3, but not IGF2BP1/2 regulates early embryogenesis by stabilizing maternal mRNA. [124] In the postimplantation embryo development process, m^6^A is indispensable and its loss will result in a hyper naïve state without entering lineage differentiation. [104] *METTL3* and/or *METTL14* KO will cause embryonic lethality via m^6^A mechanisms, which is also observed in *METTL16* or *YTHDC1* KO mice. [104,118,125] Taken together, the m^6^A-related proteins (writers, readers and erasers) have been shown significant roles in stem cell proliferation/self-renewal/reprogramming and embryonic development, however, the full understanding of m^6^A-dependent and independent mechanisms remains to be further discovered.

### 4.6. Neuro differentiation

*Mettl14* cKO in nervous system prolonged the cell cycle of neural stem cells (NSCs), leading to the cortical neurogenesis extending into postnatal stages and further brutal postnatal death. [126] Another study showed that *Mettl14* deletion also induced postnatal death by reducing proliferation and premature differentiation using the same cKO mouse. [127] *Ythdf2* deletion impairs self-renewal and spatiotemporal neurogenesis of NSCs, leading to lethality at late embryonic stages. [128] *Fmr1* deficiency extended maintenance of NSCs due to a delayed cell cycle progression and differentiation by inhibiting neural differentiation-related mRNAs export as an m^6^A reader protein. [129] *Mettl3* KO results in cerebellar hypoplasia by increasing the m^6^A-modified apoptosis- or cerebellar development-related mRNAs. [130] *Alkbh5* deletion in mice exposed to hypobaric hypoxia regulates abnormal proliferation and differentiation by regulating m^6^A-modified mRNAs export process. [131] Moreover, *FTO* controls axon growth by regulating the local translation of m^6^A-modified GAP-43 mRNA. [132] *Ythdf1* maintains axon guidance by promoting *Robo3.1* mRNA translation via an m^6^A-dependent manner. [133] *Mettl14* deletion delays the injury-induced translation of regeneration-associated genes and thus impairs axon regeneration and function recovery via *Ythdf1*-m^6^A-*Atf3* axis in adult nervous system. [134] *YTHDF1* or *YTHDF3* KD compromises synaptic transmission by reducing PSD95 clustering and *GluA1* expression in cultured hippocampal neurons in an m^6^A-dependent manner. [135] *Fto* deficiency inhibits the proliferation and neuronal differentiation of adult NSCs. [136,137] On the contrary, *Mettl3* deletion also reduces adult NSCs proliferation by decreasing Ezh2 and H3K27me3 levels. [138] The underlying dedicated molecular mechanism of *Fto*- and *Mettl3*-regulated m^6^A in regulating adult NSCs proliferation and differentiation required further investigations. It would be interesting to evaluate the potential roles of Fto or Mettl3 in m^6^A-independent manners in adult NSCs. In contrast, *METTL14* KO inhibits proliferation but promotes differentiation of embryonic neural stem cells by increasing the abundance of H3K4me3, H3K27me3 and H3K27ac. [127] Although METTL3 and METTL14 are two proteins, their functions are similar in most contexts or cells, because they usually form an MTC when depositing m^6^A, and the protein abundance will be immediately downregulated once either of them is inhibited. Again, the m^6^A-independent mechanism warrants further investigation. In the embryonic cortical neurogenesis, *METTL3* and *METTL14* KO enhance the cell cycle of cortical neural progenitor cells and block differentiation of radial glial cells. FMRP and Prcc2a, two newly identified m^6^A reader proteins, play essential roles in regulating neural progenitor cell proliferation and oligodendrocyte development, respectively.

*Mettl14* deletion in developing or postmitotic oligodendrocytes results in significant reduction of mature oligodendrocytes and hypomyelination by regulating the splicing of target m^6^A-modified mRNAs such as *Ptprz1* and *Nf155*. [139] *Prrc2a* has been identified as another m^6^A reader protein and its deletion in oligodendrocyte progenitor cells induces locomotive and cognitive defects by regulating m^6^A-modified *Olig2* mRNA. [35].

It has been reported that synaptic FTO inhibits memory formation and increased m^6^A abundance due to the decreased FTO expression facilitates the experience-induced memory or fear memory consolidation. [140,141] Consistently, METTL3 deletion will decrease the formation of hippocampus-dependent long-term memory and METTL3 overexpression facilitates long-term memory consolidation, by promoting the translation of m^6^A-modified target mRNAs involved in memory formation such as *Arc*, *Egr1*, *c-Fos*, *Npas4* and *Nr4a1*. [142] Moreover, YTHDF1 is found to be essential for the m^6^A-enhanced learning and memory in the hippocampus by translocating into the postsynaptic density fraction and promoting m^6^A modified mRNA translation. [143] Taken together, this is a rare but good example to show a consistent role of m^6^A modification in the process of memory formation by understanding the functions of its writer, reader or eraser proteins such as METTL3, YTHDF1 and FTO.

### 4.7. Senescence

Senescence is widely observed in aging and diseased tissues where pro-inflammatory cytokines are secreted and further form a special microenvironment called senescence-associated secretory phenotype (SASP). [144] RNA m^6^A methylation abundance is found much less in elderly PBMC compared to that in young PBMC. [145] METTL3 deletion induces a premature senescence phenotype in human bone marrow mesenchymal stem cells (hMSC) by destabilizing *MIS12* mRNA in an IGF2BP2/m^6^A-dependent manner. [146] Controversially, another group identified that m^6^A modification sites/abundance increased in both elderly mice and human brains compared to the young brains. [147] However, it is surprising that a systematic study revealed that the genome-wide redistribution of METTL3 and METTL14 at the enhancer and promoter regions drives the SASP in an m^6^A-independent mechanism. [148] It is complicated and difficult to make a conclusion about the roles of m^6^A modification in cellular senescence and aging processes, distinct conclusions made in each study may be due to the different contexts (e.g. MSC vs PBMC vs brain tissues). Whether the modification related proteins play essential roles relied on their enzyme activities required further and careful investigations.

### 4.8. LLPS formation

Cells usually can be divided into several compartments or subcellular structures with distinct dedicated roles such as membrane-separated organelles or membraneless organelles which are driven by liquid-liquid phase separation (LLPS). [149,150] Notably, non-covalent interactions between RNA binding proteins and nucleic acids serve as driving forces for LLPS and the formation of membrane-less organelles. [151] It is found that RNA m^6^A modification has become a key regulator of LLPS formation in recent years. For example, YTHDF family proteins (YTHDF1/2/3) can form the LLPS in vitro and cells, and this formation can be enhanced by multiple sites (but not single) with m^6^A modification. [42] The capacity of forming LLPS by m^6^A-modified RNAs and YTHDF2 complex is also validated in another study, [152] so does YTHDF1. [153] Moreover, it has also been found that m^6^A-modified mRNAs promote YTHDF1 and YTHDF3 induced formation of stress granules, where a membraneless organelle driven by LLPS. [154] Consistently, m^6^A binding domain of YTHDF3 promotes its LLPS formation and promotes AR mRNA translation in prostate cancer. [155] YTHDF2 forms LLPS with TDP43 and promotes RNA destabilization in amyotrophic lateral sclerosis (ALS). [156] Interestingly, YTHDF1 is located in stress granules and forms LLPS to enhance *CLOCK* mRNA translation in Asthma. [157] FMR1, a new m^6^A reader protein, has been found to form LLPS by interacting with m^6^A-modified mRNAs and in turn promotes their degradation. [158] MTC component METTL3 has also been shown can form LLPS itself, which is critical for m^6^A regulation. [159] It is also shown that the phosphorylation of METTL14 at Thr72 promotes its LLPS and further enhances m^6^A modification of Nox2. [49] RBM15, as another component of MTC, was also shown to form LLPS in the deposition of m^6^A modification on target mRNAs such as STYK1 and further stabilize/promote its expression. [160] Moreover, YTHDC1 interacts with m^6^A-modified eRNA in nucleus and facilitates LLPS by enhancing transcription activator BRD4 condensates to further promote gene transcription. [83] Intriguingly, YTHDC1 binds to and promotes the export process of m^6^A-modified nuclear mRNAs by forming LLPS-mediated condensates, which protects the modified target mRNAs from PAXT complex degradation in leukemogenesis. [161] IGF2BP1 also forms LLPS based on its m^6^A binding domain, KH3–4 domain, which process is promoted by interacting with m^6^A-modified targets such as *HPV E7* or *RUNX-IT1* in malignant contexts. [162,163] It is obvious that the RNA binding proteins especially m^6^A reader proteins and writer proteins can form LLPS in either normal or malignant contexts. Moreover, the m^6^A-modified RNAs facilitate this process, indicating that the membranless condensates are critical/required for RNA modifications. Besides the writer and reader proteins of m^6^A, whether eraser proteins can form LLPS has not yet been investigated.

## 5. Immune responses

### 5.1. Innate immune responses

**Fig. 4.**
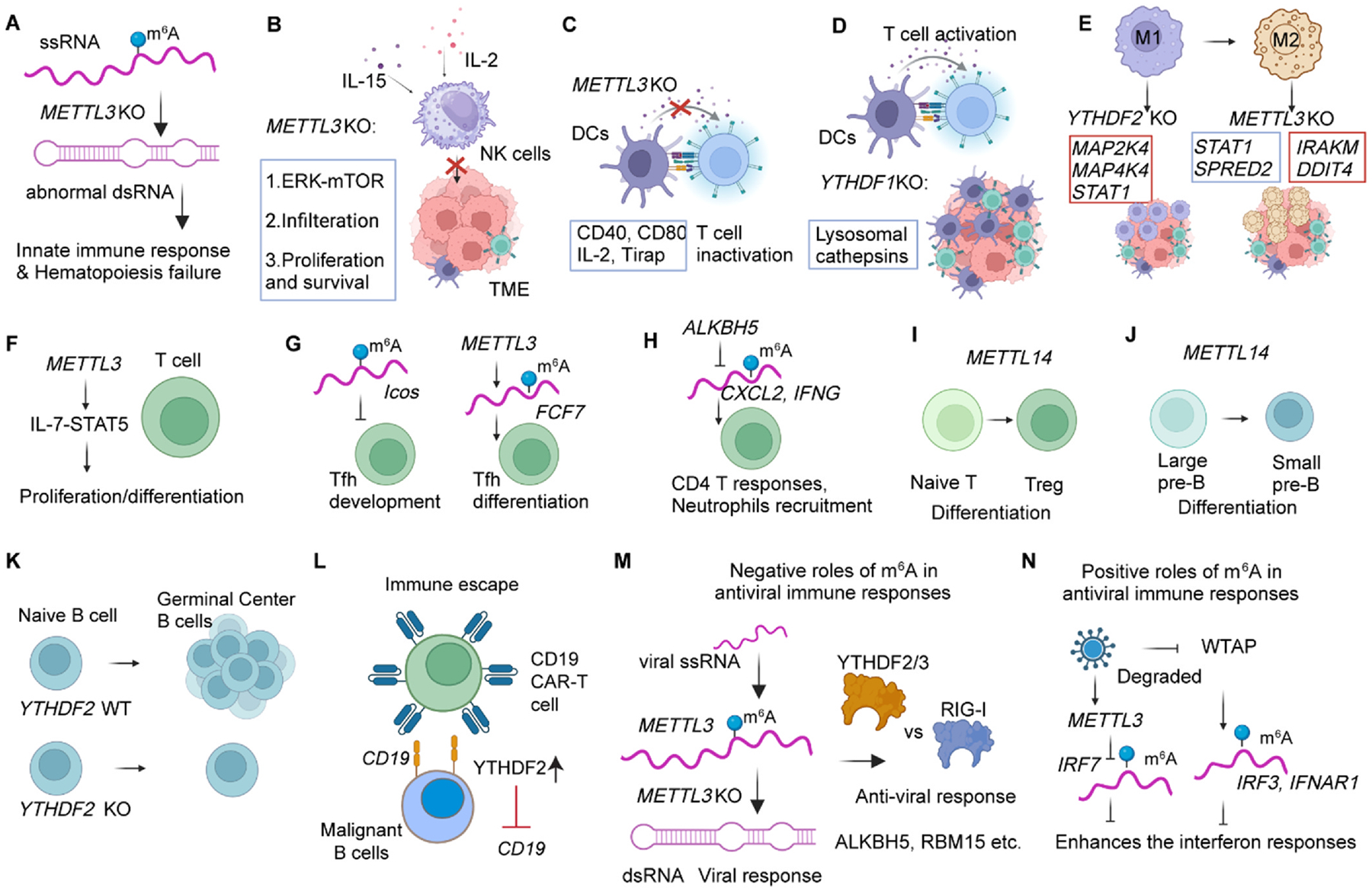
The roles of m^6^A and its related proteins in innate immune responses and adaptive immune responses. (A-E) Alterations of innate immune responses after changing m^6^A abundance in immune cells including NK cells (B-C), DCs (D) and macrophages (E). (F-L) The change of adaptive immune responses when altering m^6^A abundance such as the change of T cell proliferation (F) T cell differentiation (G-I) or B cell differentiation/proliferation and B cell immune escape (J-L). (M-N) The roles of m^6^A during antiviral immune responses.

*Mettl3*-deficient HSCs results in a failure of hematopoiesis due to aberrant activation of an innate immune response by increasing formations of double-stranded RNAs (dsRNAs) and the related RNA-sensing pathways such as OAS-RNase L, PKR-eIF2a, MDA5 and RIG-I. Abnormal innate immune response and increased dsRNA have also been observed in intestinal epithelial cells upon *Mettl3* deletion (Fig. 4A). [164].

#### 5.1.1. NK cells

NK cells’ natural cytotoxicity and cytokine production are critical for the innate immune response. *Mettl3* deficiency results in the decreased infiltration and function of NK cells in tumor microenvironment thus promoting tumor growth and shorter survival time in mice by decreasing the protein expression of m^6^A-modifed SHP-2 mRNA, which leads to a suppressed activation of AKT and MAPK signaling pathway in NK cells (Fig. 4B). [165] It is also reported that METTL3 deletion impairs NK cells responsiveness to IL-15 by decreasing the expression of PTPN11 (Fig. 4B). [166] It seems consistent that m^6^A is positively related to NK cell growth, survival and functions, which may be due to the limited publications in this area. Whether other m^6^A related proteins such as erasers (FTO and ALKBH5) and METTL16 (another writer protein) play consistent roles merit more investigations.

#### 5.1.2. Dendritic cells (DCs)

DCs are key antigen-presenting cells that initiate immune responses by activating T cells in both the innate and adaptive immune systems. METTL3 is essential for maturation and promotes DCs activation and DCs-mediated T cell activation by maintaining m^6^A-modified CD40, CD80 and the toll-like receptor (TLR) signaling, which are recognized and mediated by YTHDF1 (Fig. 4C). [167] However, another report found that m^6^A exerts a negative regulatory role in antitumor immune responses. [168] YTHDF1 promotes the translation of lysosomal proteases (m^6^A-modified), which degrade tumor neoantigens in DCs, and further immune escape from infiltration and killing of CD8^+^ T cells (Fig. 4D). [168] Similarly, it is reported that DCs produce fewer cytokines and activation markers when treated with m^6^A-modified RNAs compared to unmodified RNAs. Therefore, the role of m^6^A in DCs activation, stimulation and maturation remains controversial and requires more studies. Comprehensive and systematic investigations are required to compare the consequences of YTHDF1-mediated CD40, CD80, TLR and proteases in DCs-mediated T cell activation in tumor microenvironment.

#### 5.1.3. Macrophages

Macrophages play critical roles in innate immune responses, including the digestion and removal of cellular debris, antigen presentation, and the secretion of cytokines to enhance host immune defense against infections. They also participate in cross-presenting antigens to kill T cells during the antitumor process. Resting macrophages can be activated and polarized to initiate stimulus-specific transcriptional programs through the engagement of pattern recognition receptors and cytokine receptors. [169] Recently, it has been reported that METTL3 deletion reduces the M1 (pro-inflammatory and antitumor activity) but stimulates M2 macrophages (immunosuppressive and protumor phenotype) by destabilizing the m^6^A-modified *STAT1* mRNA, which is a critical controller in M1 polarization (Fig. 4E). [170] Similarly, another paper also revealed that METTL3 deficiency inhibited the toll-like receptors (TLRs) signaling-mediated pro-inflammatory cytokines production and macrophage activation by increasing *IRAKM* mRNA (a negative regulator of TLRs) in an m^6^A manner, resulting in enhanced tumor growth and reduced TNF-α secretion (Fig. 4E). [171] Intriguingly, a recent report found that METTL3 deletion in macrophages significantly increases M1/M2-like tumor-associated macrophages and regulatory T cell infiltration into tumors, thus promoting tumorigenesis and metastasis, by inhibiting YTHDF1-mediated translation of *SPRED2* and further enhancing the expression of TNF-α, IL-6 and ARG1. [172] Another report also found that the loss of METTL3 reduces mTOR and NF-κB signaling activities by stabilizing/increasing DDIT4 transcripts involved in metabolic endoplasmic reticulum stress and TNF-α stimulation (Fig. 4E). [173] Additionally, METTL14 enhances the translation of m^6^A-modified target mRNA SOCS1, which further inhibits TLR4/NF-κB signaling. [174] Moreover, YTHDF2 deletion significantly promotes the pro-inflammatory responses of macrophages by increasing m^6^A-modified MAP2K4 and MAP4K4 mRNAs and further increasing downstream cytokines secretion such as TNF-α, IL-6, etc. (Fig. 4E). [175] Similarly, YTHDF2-deficient macrophages polarize to M1 antitumor phenotype and enhance antigen cross-presentation ability to CD8^+^ T cells, thus inhibits the tumor growth and progression in tumor microenvironment (Fig. 4E). [176] Taken together, the role of METTL3/METTL14-mediated m^6^A in tumor microenvironment and progression has been shown consistent, however, the mechanisms such as polarization phenotype upon METTL3 deletion in macrophages, change of same signaling (NF-κB, TNF-α secretion, etc.) is not that consistent under different conditions or contexts. More and more studies in similar conditions are required to fully understand the role of RNA m^6^A modification in macrophages in immune responses. Whether other writer proteins such as METTL16 and eraser proteins such as ALKBH5 or FTO play similar and consistent role in macrophage function remain elusive and merit further investigation.

### 5.2. Adaptive immune responses

#### 5.2.1. T cells

T cell is a type of immune cells involved in adaptive immune response. It has been reported that METTL3-mediated m^6^A modification induces degradation of STAT signaling inhibitory genes *Socs* mRNAs, and thus maintains the IL-7-mediated STAT5 activation and T cell homeostatic proliferation/differentiation (Fig. 4F). [177] The loss of Mettl3 in mice leads to the failure of homeostatic expansion and block in naïve state for up to 12 weeks. [177] It is also found that low m^6^A leads to a loss of suppressive functions by increasing SOCS activity and inhibiting IL2-STAT5 pathway. [178] The initiation of follicular helper T (Tfh) development is also suppressed by m^6^A modification on the essential *Icos* mRNA (Fig. 4G). [179] METTL3 deletion results in defects in the differentiation of Tfh cells by destabilizing FCF7 mRNA in an m^6^A manner (Fig. 4G). [180] ALKBH5 loss leads to reduced severity of T cell-regulated colitis by increasing *CXCL2* and *IFNG* via an m^6^A-dependent mechanism (Fig. 4H). [181] It has also been found that METTL14 deletion inhibits the differentiation of naïve T cells to Treg cells (Fig. 4I). [182] The functions and mechanisms of m^6^A and its related proteins in T cell subsets differentiation and proliferation are largely unknown and merit further investigations.

#### 5.2.2. B cells

B cells, as another key component of adaptive immune response, are essential for antibody production and immune responses. It has been reported that METTL14 deletion represses the proliferation of IL-7-mediated progenitor B cells and differentiation from large precursor B cell to small precursor B cells (Fig. 4J). [183] Interestingly, recent studies showed that YTHDF family proteins are highly expressed in B cells in the seeding processes of pre-germinal center formation, and *Ythdf2* KO inhibited the germinal center responses by reducing germinal center B cells (Fig. 4K). [184] Notably, previous reports have shown that m^6^A is most abundant in B cells compared to other cell types such as T cells, macrophages, etc. [56] However, the role of m^6^A machinery proteins in normal B cell development as well as in malignant B cells remains largely unknown. Our recent data identified that YTHDF2-m^6^A-CD19 mediated malignant B cell immune escape from CD19 CAR-T cell therapy, suggesting that YTHDF2 serves as a promising therapeutic target for malignant B cells including B cell leukemia or lymphomas (Fig. 4L). [38,185] The underlying mechanism of m^6^A abundance in B cell contexts remains to be further investigated.

### 5.3. Antiviral immune responses

In 1970s, m^6^A marks in viral RNA were first identified and found, [186,187] however, no m^6^A methyltransferases were found in viral genome to date, [188] indicating that viral m^6^A-RNA must be deposited by host writer proteins. Many groups have shown that writer proteins significantly increased or decreased erasers upon virus infection. [189–191] An ideal hypothesis is that virus take use of the host’s enhanced writer proteins to deposit m^6^A marks in viral RNA mimicking host RNA and thus further suppresses type I interferon production and host innate immune over activation. Indeed, METTL3 has also been found to reduce viral dsRNA formation and thus inhibit antiviral immune signaling by depositing m^6^A on viral RNA (Fig. 4L). [189] It is also reported that YTHDF2 and YTHDF3 compete with RIG-I (host dsRNAs sensor protein) to bind with m^6^A modified viral RNAs (Fig. 4L). [192,193] Moreover, m^6^A sites mutation or removal by overexpressed eraser protein can trigger RIG-I-dependent type I interferon stimulation/activation. [192–196] It was also reported that antiviral innate immune responses are suppressed by the host RNA m^6^A modifications such as type I interferons IFN-β. [190] Another example is that METTL3 is increased upon hepatitis B virus infection and further modifies and reduces PTEN mRNA, which can promote the IRF3 export to cytoplasm to initiate interferon synthesis. [197] IKKγ and p65 can be degraded by the axis of YTHDF2-DDX5-m^6^A, which in turn suppresses the antiviral innate immune responses. [198] The loss of METTL3 or YTHDF2 results in a significant increase of interferon-stimulated genes following a viral infection or stimulation by an inactivated virus. [190] Moreover, YTHDF3 inhibits interferon-dependent antiviral response by promoting the translation of FOXO3, which represses INF response, in an m^6^A manner. [199] Multiple reports also have found that METTL3, RBM15 and YTHDF2 show a pro-viral and suppressive role of innate immune response upon virus infection (Fig. 4L). [200–203] In addition, ALKBH5 is found to decrease upon virus infection, leading to a reduction of α-ketoglutarate dehydrogenase OGDH in an m^6^A manner and subsequent reduced metabolite itaconate production, which is vital for viral replication (Fig. 4L). [191] These studies consistently showed that m^6^A is a negative regulator in antiviral immune responses. Interestingly, another study also reported that the scaffold protein of MTC, WTAP, is degraded and thus results in inhibition of innate immune responses by reducing m^6^A abundance and subsequently decreasing translation of IRF3 and IFNAR1 mRNA upon virus infection (Fig. 4M). [204] IRF7 mRNA is decayed by METTL3-mediated m^6^A modification and further enhances the interferon responses to protect from rotavirus infection (Fig. 4M). [205] Additionally, DDX46 suppresses the type I interferon by altering the export of antiviral transcripts from nucleus to cytoplasm via recruiting m^6^A eraser protein ALKBH5, which avoids over activation of innate antiviral responses, suggesting m^6^A modification is a positive regulator of antiviral immune responses. [206] Taken together, most of the current studies identify the pro-viral and suppressive role of either viral m^6^A modification or host m^6^A marks in the process of host antiviral innate immune responses, suggesting a potential approach in treating virus infection.

## 6 m^6^A modification in cancer developments

6.

### 6.1. Oncogenic m^6^A modifiers

#### 6.1.1. METTL3

**Fig. 5.**
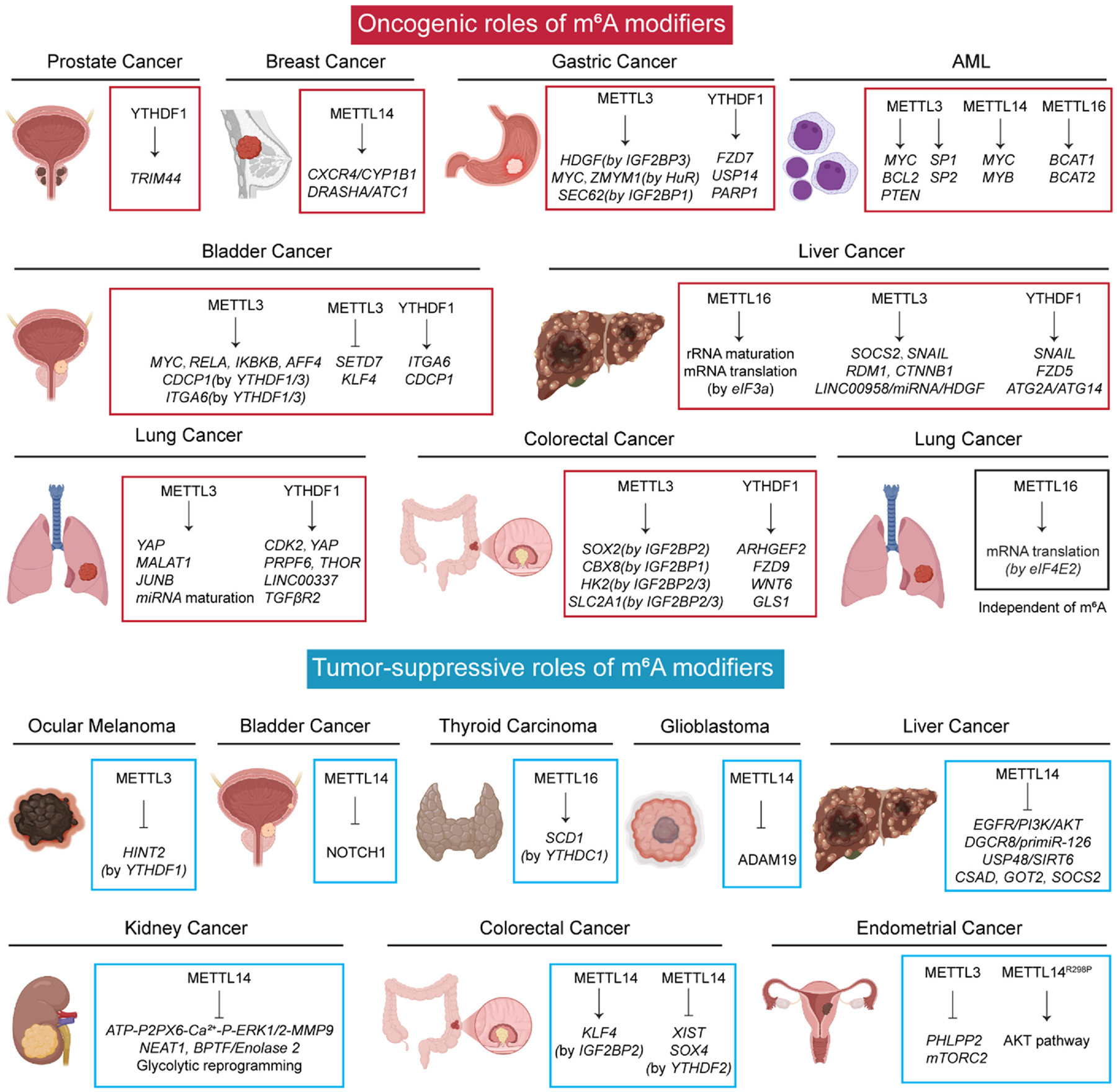
Comprehensive roles of m^6^A proteins in different types of cancers. (Red frames indicate oncogenic roles, blue frames indicate tumor suppressive roles).

METTL3 abundance is much higher in AML cells compared to normal HSPCs and its deletion induces cell differentiation/apoptosis, cell cycle arrest and delayed onset of leukemia in vivo. [109,207] Mechanically, METTL3 promotes the translation of target mRNAs such as *MYC, BCL2* and *PTEN* or binds to the promoter region and further activates the transcription of *SP1* and *SP2* mRNAs in an m^6^A-dependent manner. [109,207] Moreover, METTL3 has also been shown overexpression and strong correlation with patient prognosis in liver cancers such as hepatocellular carcinoma and hepatoblastoma through m^6^A-dependent manners by determining the fates of different target genes such as *SOCS2, SNAIL, RDM1*, LINC00958-miRNA-HDGF and *CTNNB1*. [208–212] *METTL3*-elevated m^6^A abundance promotes glycolysis, cell growth and metastasis by promoting IGF2BP3-mediated *HDGF* mRNA stability in gastric cancer cells. Consistently, other groups discovered other target genes of METTL3-m^6^A such as *MYC* target genes, *ZMYM1* (stability is enhanced by HuR) and *SEC62* (stability is enhanced by IGF2BP1) in the cell proliferation and EMT progression in gastric cancer. [213–217] METTL3 has also been found to show high expression in metastatic tissues and association with prognosis in patients with colorectal cancer. Three different studies identify different m^6^A-dependent mechanisms and related target gene mRNAs such as IGF2BP2-stabilized *SOX2*, IGF2BP1-stabilized *CBX8*, and IGF2BP2/3-stabilized *HK2* and *SLC2A1* in colorectal cancer cells. [218–220] In lung cancer, METTL3 and m^6^A abundance are found increased in non-small cell lung cancer cells during the EMT or metastasis and drug resistance progression. Deletion of METTL3 induced the downregulation of target m^6^A-modified mRNAs such as *YAP*, *MALAT1*, *JUNB* and even premiRNA maturation. [102,221,222] METTL3 also plays oncogenic roles in bladder cancer by either promoting the expression of *AFF4, IKBKB, RELA, MYC*, or promoting YTHDF1/3-regulated translation of m^6^A-modified *CDCP1* and *ITGA6,* or even by repressing the tumor suppressor genes such as *SETD7* and *KLF4*. [223–226] In addition, METTL3 has shown an elevated expression and oncogenic roles in many other cancers such as pancreatic cancer, glioblastoma, prostate cancer, breast cancer, osteosarcoma, oral squamous cell carcinoma, thyroid carcinoma, and head and neck squamous cell carcinoma by regulating different target genes in m^6^A-dependent manners (Fig. 5).

#### 6.1.2. METTL14

It has been reported that METTL14 abundance is elevated in pancreatic cancer samples and promotes tumor cell growth/proliferation, migration, invasion and colony formation by the m^6^A-dependent mechanisms. [227,228] METTL14 has also been shown to play an oncogenic role in breast cancer by promoting CXCR4/CYP1B1 or DRASHA/STC1 axis in m^6^A manner. [229,230] Notably, METTL14 promotes both normal HSPCs and AML stem cells by enhancing the mRNA stability and translation of m^6^A-modified mRNAs such as MYC and MYB (Fig. 5). [110].

#### 6.1.3. METTL16

The METTL16 abundance has been found increased in cholangiocarcinoma tissues and its deficiency inhibits cell proliferation and tumor progression by reducing YTHDF1-mediated translation process of target mRNA PRDM15. [231] METTL16 has also been shown to play an oncogenic role in hepatocellular carcinoma by enhancing ribosomal RNA maturation and mRNA translation via interacting with eIF3a, but whether this process more relies on m^6^A modification or not requires further investigations. [232,233] Moreover, METTL16 is highly expressed in AML cells, especially leukemia stem/initiating cells, and its deletion inhibits the growth and progression of AML cells in mice via METTL16/m^6^A/BCAT1–2/BCAA axis (Fig. 5). [234].

#### 6.1.4. YTHDF1

It is consistent that YTHDF1 is always highly expressed and plays oncogenic roles by enhancing translation in m^6^A manners in multiple different types of cancers. For example, YTHDF1 loss significantly suppresses gastric cancer cell proliferation (via inhibiting m^6^A-modified FZD7 mRNA translation)^25^, metastasis (via m^6^A-modified USP14)^171^, and oxaliplatin resistance (via m^6^A-modified PARP1) [235]. YTHDF1 also plays essential and oncogenic roles in colorectal cancer progression and metastasis via an m^6^A-dependent manner by regulating different target mRNAs translation such as *ARHGEF2*, [236] *FZD9*, [237] *WNT6* [237] and *GLS1* [238]. YTHDF1 promotes liver cancer cell proliferation, metastasis, and autophagy by regulating the translation of m^6^A-modified mRNAs such as *Snail, FZD5, ATG2A* and *ATG14*. [181,239,240] Similar in lung cancer, YTHDF1 promotes cell growth, invasion, and EMT and cisplatin resistance of NSCLC cells by regulating the translation of *CDK2, YAP* and *PRPF6* or by regulating RNA stability of lncRNA THOR, LINC00337 and TGFβR2 in an m^6^A manner. [241–246] Knockdown of YTHDF1 suppresses prostate cancer cell proliferation, migration, invasion, lymph node metastasis by targeting m^6^A-modified *TRIM44*. [247,248] YTHDF1 is also found highly expressed in bladder cancer and it promotes the cell growth and progression by enhancing translation of m^6^A-marked *ITGA6* and *CDCP1*. [224,225] Consistently, the abundance of YTHDF1 is widely overexpressed in multiple other cancer types, such as ovarian cancer, breast cancer and osteosarcoma, and plays oncogenic roles by regulating different target genes. [249–254] Therefore, YTHDF1 widely promotes tumorigenesis and mRNA translation in an m^6^A manner in cancers, whereas the tumor suppressive functions or mRNA stability regulation were rarely reported (Fig. 5).

### 6.2. Tumor-suppressive m^6^A modifiers

#### 6.2.1. METTL3

Although many studies identified the oncogenic roles of METTL3 in many cancers, some groups showed the opposite phenotypes of METTL3 in the same cancer types with a distinct mechanism. For example, a novel statistical analysis found that METTL3 is a tumor suppressor gene and its somatic mutations promote bladder cancer cell growth/proliferation. [255] Moreover, METTL3 deficiency promotes the glioblastoma progression by regulating self-renewal of glioblastoma stem cells. [256] In colorectal cancer, METTL3 suppresses cell proliferation/migration/invasion in an m^6^A-dependent manner. [257] In ocular melanoma, METTL3 is downregulated and promotes the YTHDF1-mediated translation process of tumor suppressive gene *HINT2*. [258] Notably, m^6^A hypomethylation caused by METTL3 low expression leads to downregulation of *PHLPP2* (negative AKT regulator) and upregulation of *mTORC2* (negative AKT regulator) in more than 70 % of endometrial tumors, suggesting a tumor suppressive role of METTL3 (Fig. 5). [259].

#### 6.2.2. METTL14

Given the broad oncogenic roles of METTL3 in cancers, it is very interesting that METTL14 exerts antitumor functions in an m^6^A-depnedent manner in most tumors, which brings our attention to understand their roles as a whole MTC. For example, in colorectal cancer, METTL14 inhibits the proliferation, metastasis and invasion of tumor cells either by enhancing the expression of KLF4 in IGF2BP2-m^6^A manner, [260] or by inhibiting YTHDF2-mediated SOX4^72^, or by suppressing XIST [261]. In liver cancer, multiple different groups’ investigations showed that METTL14 inhibits EMT, invasion and metastasis of liver cancer cells by regulating m^6^A-modified target mRNAs such as EGFR/PI3K/AKT, DGCR8/primiR-126, USP48/SIRT6/glycolysis, CSAD, GOT2 and SOCS2. [97,124,262,263] METTL14 has been shown to be downregulated in patients with breast cancers and negatively correlates with tumor grade. [264–266] R298P mutation of METTL14 activates the AKT pathway and promotes the proliferation and migration of endometrial cancer cells in an m^6^A manner. [259] METTL14 is widely found lowly expressed in bladder cancer cells and tumor initiating cells, and its deletion promotes the development of bladder cancer by increasing NOTCH1 expression. [267] Moreover, increased METTL14 expression upon isorhapontigenin treatment facilitates the migration, invasion and EMT of bladder cells. [268] Deletion of METTL14 promotes the m^6^A-modified ADAM19, resulting in the self-renewal and tumorigenesis of glioblastoma stem cells. [256] In kidney cancer, METTL14 is also widely downregulated in cancer cells compared to normal kidney tissues. [269] METTL14 deletion promotes the kidney cancer cell growth, proliferation, migration and invasion either by enhancing the signaling pathway of ATP-P2PX6-Ca^2+^-P-ERK1/2-MMP9 [270], or by increasing NEAT1 [271] or by increasing BPTF/enolase 2 abundance and glycolytic reprogramming [272]. METTL14 SNP mutation is correlates with reduced risk of pediatric ALL patients. [273] Moreover, low expression of METTL14 correlates with poor prognosis in many other cancer cells such as adenocarcinoma, prostate cancer, breast cancer, gastric cancer, osteosarcoma etc. (Fig. 5). [247,274–276].

#### 6.2.3. METTL16

METTL16 is down-regulated in papillary thyroid carcinoma and inhibits the tumorigenicity through m^6^A/YTHDC1/SCD1-regualted lipid metabolism. [277] Pancreatic ductal adenocarcinoma (PDAC) with highly expressed METTL16 is more sensitive to poly-(ADP-ribose)-polymerase inhibitor (PARPi) treatment, especially when combined with gemcitabine, [278] with a consistent phenotype as observed by others in PDAC. [279–281] Moreover, a similar role of METTL16 is observed in bladder cancer and ovarian cancer. [282,283] Whether METTL16 plays a tumor-suppressor role in other types of cancer merits further investigations.

### 6.3. Roles of modifiers in cancers independent of m^6^A

#### 6.3.1. METTL3

It has been reported that METTL3 binds to about 22 % of all m^6^A modified sites in transcriptome, indicating the non-m^6^A related mechanism remains crucial in cancer cells. [11] It has been found that METTL3 promotes the translation initiation of congenic genes such as *EGFR* and *TAZ* by recruiting eIF3 independent of its catalytic activity in human lung cancers. [284] Furthermore, the research group also identified that METTL3 N terminal interacts with eIF3 subunit h and control the translation initiation in lung cancers independent of m^6^A. [285] In gastric cancer, METTL3 has also been found to bind to non-m^6^A-modified target mRNAs and further promotes their translation by interacting with eIF4F (Fig. 5). [286].

#### 6.3.2. METTL16

Recent studies have unveiled a novel, m^6^A-independent role of METTL16 that promotes translation and tumorigenesis by interacting with eIF4E2 in lung cancer independent of m^6^A catalytic activity. [287] In lung cancer cells, METTL16 is overexpressed and predominantly localized in the cytoplasm, where it directly interacts with eIF4E2. This interaction sequesters eIF4E2, preventing it from binding to the 5’ cap structure of mRNAs, thereby facilitating cap-dependent translation. The enhanced translation of oncogenic mRNAs contributes to increased tumor cell proliferation and tumorigenesis (Fig. 5). Moreover, METTL16 also interacts with MRE11 in an RNA-dependent manner, and this interaction inhibits MRE11’s exonuclease activity in a methyltransferase-independent manner in PDAC. [278] It is also reported that METTL16 directly interacts with eukaryotic initiation factor 3a/b (eIF3a/b) and ribosomal RNAs (rRNAs) through its Mtase domain, promoting the assembly of the translation initiation complex (TIC) and subsequent translation of over 4000 mRNA transcripts in liver cancer stem cells. [232,233].

It is interesting and required to study whether a specific m^6^A modifier work in an m^6^A-dependent or -independent manner during the carcinogenesis and progression. Given the dural mechanisms of m^6^A-related proteins, it would be also critical to investigate which mechanism is the major one in biological and pathological processes, providing a more straightforward path for developing pharmacological inhibitors.

### 6.4. The discrepancy of m^6^A-dependent mechanisms in cancers

The context-dependent roles of m^6^A modifiers in cancers can be attributed to several interrelated factors. Firstly, the biological effects of m^6^A regulators such as METTL3 and METTL14 vary significantly across different cancer types, as these enzymes may promote tumor progression in one context while acting as tumor suppressors in another. [288] This discrepancy is partly due to their differential subcellular localization, where METTL3, for instance, can enhance mRNA translation in the cytoplasm but modulate splicing or stability in the nucleus. [289] Furthermore, certain m^6^A modifiers exert their functions independently of m^6^A catalytic activity, suggesting alternative mechanisms such as direct interaction with translation initiation complexes. [289] The tumor microenvironment also plays a critical role, as the expression of m^6^A enzymes has been associated with altered immune cell infiltration, potentially influencing immune evasion. [153] Additionally, mutations in m^6^A-related genes, like the R298P mutation in METTL14, may alter RNA substrate recognition, leading to dysregulated gene expression. [290] These factors collectively contribute to the diverse and some-times opposing roles of m^6^A modifiers in different cancer contexts.

For the same cancer context but with opposite functions of m^6^A modification, it may be explained by the distinct protein/signaling pathway and the different m^6^A-modified gene sets. Therefore, m^6^A modification’s role may vary depending on which target/signaling pathways were studied, which should be described precisely and carefully. For the same protein/signaling pathway in one cancer context but with distinct roles of m^6^A modification, it is possible that the protein plays a critical role under an m^6^A-independent mechanism more than the m^6^A-dependent mechanism, which derived from its catalytic activity or binding capacity. It may require a systematically study to compare which mechanism contribute more in the specific context before making a conclusion.

## 7. Antitumor immunity

### 7.1. Tumor intrinsic m^6^A related proteins

Deletion of ALKBH5 enhances the m^6^A abundance on target mRNAs such as PD-L1 and thus further destabilizes PD-L1 mRNA and facilitates T cell antitumor immunity. [291] METTL3 or METTL14 deletion in tumor cells increases STAT1 and IRF1 mRNA abundance in an m^6^A-decay mechanism, and thus further elevates the CXCL9 and CXCL10, two essential chemokines can recruit the effector T cells or NK cells expressing CXCR3 into tumor, indicating that m^6^A-mediated chemokines contribute immune cell infiltration. [292] ALKHB5-deficient glioblastoma cells decrease tumor associated macrophages and increase CD8^+^ T cells infiltration, by increasing the chemokine CXCL8/IL8 in an m^6^A manner. [293] Another study also showed that ALKBH5 deletion decreases infiltration of myeloid-derived suppressive cells (MDSCs) by destabilizing MCT4/SLC16A3. [294] MHC-I expression and interferon expression are significantly increased upon YY1-CDK9 suppression by reducing m^6^A abundance through repressing METTL3 and YTHDF2 expression, leading to the enhanced immunotherapy efficacy. [295] Moreover, T cells are activated upon FTO deletion by reducing the glycolytic activity in tumor cells, which in turn facilitate the recognition and killing efficacy. [296] However, the detailed mechanism of m^6^A modification and its related proteins under the infiltration and killing of the immune T cells, NK cells and even other immune cells in antitumor process largely remains unclear.

### 7.2. Immune cells in tumor microenvironment

It has been reported that METTL3 promotes the antitumor processes in NK cells. The loss of METTL3 in NK cells significantly reduces the cytotoxic effect compared to wild type NK cells. [165] YTHDF2 deletion impairs the IL-15-STAT5-mediated NK cell survival and antitumor immunity. [297] It remains elusive whether this mechanism exists in other IL-15-mediated antitumor immune cells such as CD8^+^ T cells. It has also been shown that METTL14 deletion in tumor-associated macrophages increases EBI3 and further cross-talks with T cells and leads to its differentiation/dysfunction, which promotes the colorectal cancer progression. [298] YTHDF1 in DCs represses the antigen cross-presentation with tumor-infiltrating CD8^+^ T cells. [168] Given the general supportive and positive roles of m^6^A writer proteins in antitumor immune cells, however, also an oncogenic roles of these proteins in some tumors, it remains an open topic to develop therapeutic drugs that targeting these proteins in specific tumors, which will result in opposite effects in an intrinsic tumors or immune microenvironment.

## 8. Translational and clinical potential in clinical oncology

Based on the critical roles of m^6^A-related proteins in tumor development, targeting these proteins has emerged as a promising strategy to treat cancer. To date, 11 different FTO inhibitors (RheIn, MO-I-500, MA, MA2, R-2HG, FB23–2, CS1, CS2, Dac51, FTO-04 and FTO-43), [296,299–306] 3 METTL3 inhibitors (STM2457, UZH2 and UZH1a), [307–309] 2 IGF2BP2 inhibitors (CWI1–2 and JX5), [71,310] 3 YTHDF2 inhibitors (CCI-38, DC-Y13 and DF-A7), [38,311,312] 4 ALKBH5 inhibitors (Ena21, Ena15, 18I, IOX1 and W23–1006), [313–315] have been developed/identified. The dynamic regulation of m^6^A modification has emerged as a promising frontier in overcoming cancer therapy resistance, with translational implications highlighted by recent preclinical studies. In hepatocellular carcinoma [316], METTL3-mediated m^6^A modification of EGFR mRNA was shown to drive lenvatinib resistance, while pharmacological inhibition of METTL3 using STM2457 significantly restored drug sensitivity across multiple murine models, suggesting its potential as a combination therapy candidate. Similarly, in triple-negative breast cancer (TNBC), [317] ALKBH5-dependent demethylation stabilizes FOXO1 mRNA, promoting chemoresistance by maintaining redox balance via SOD2 upregulation. Targeting this axis through FOXO1 inhibition reversed doxorubicin resistance in vivo, underscoring the therapeutic relevance of m^6^A erasers.

Despite these advances, challenges persist in clinical translation. The pleiotropic roles of m^6^A regulators across tissues raise concerns about off-target effects, necessitating tissue-specific delivery systems. Additionally, the complexity of m^6^A-mediated resistance networks, exemplified by cross-talk between METTL3 and ALKBH5, demands precise combinatorial strategies to avoid compensatory mechanisms. While early-stage inhibitors like STM2457 show promise, optimizing their pharmacokinetics and validating biomarker-guided patient stratification remain critical hurdles. These studies collectively highlight the need for deeper mechanistic insights into context-dependent m^6^A functions and innovative approaches to harness epitranscriptomic modulation safely in clinical oncology.

## 9. Limitations and perspectives of clinical trial targeting m^6^A modifiers

Targeting m^6^A modifiers has emerged as a promising strategy in cancer therapy, given the critical roles of m^6^A modifications in tumorigenesis and progression. However, clinical trials focusing on m^6^A modifiers face several limitations and challenges that need to be addressed to fully realize their therapeutic potential. The epigenetic landscape, including m^6^A modifications, is highly complex and context-dependent. m^6^A modifications also have dual roles, acting as oncogenic drivers in some cancers while serving as tumor suppressors in others. This complexity complicates the development of universally effective therapeutic strategies targeting m^6^A modifiers. Moreover, the structural similarities among family proteins pose challenges in achieving selectivity when targeting m^6^A modifiers. Therefore, advancing the design of highly selective small-molecule inhibitors for m^6^A-related proteins is crucial to minimize off-target effects and enhance therapeutic efficacy.

## Figures and Tables

**Table 1:** Summary of m^6^A Profiling Technologies.

Technology	Resolution	Input Requirements	Detection Scope	Advantages	Limitations
MeRIP-seq	100–200 nt	5 μg mRNA	Transcriptomic m^6^A peaks	High sensitivity; widely used	Low resolution; antibody bias
miCLIP/m^6^A-CLIP-seq	Single base	1–10 μg RNA	Single base m^6^A sites	High specificity; motif-agnostic	Low throughput; technical complexity
MAZTER-seq & m^6^A-REF-seq	Single base	100 ng mRNA	~ 25 % of m^6^A sites	Low input; cost-effective	Limited to DRACH motifs
DART-seq	Single base	10 ng total RNA	YTH domain-bound m^6^A	Ultra-low input; in situ detection	Limited to YTH domain-bound sites
m^6^A-SAC-seq	Single base	30 ng total RNA	~ 65 % of MeRIP-seq	Low input	GAC motif more than AAC, cover ~65% of transcripts identified by antibody-based method, rely on recombinant DjDim1 which is not commercially available so far
m^6^A-LAIC-seq	100–200 nt	5 μg mRNA	Transcriptomic m^6^A peaks	Full-length transcript level	Low resolution; antibody bias
GLORI	Single base	200 ng mRNA	Transcriptomic m^6^A peaks	Unbiased information	High cost; unable to tell m^6^Am and m^1^A
eTAM-seq	Single base	50 ng mRNA	Transcriptomic m^6^A peaks	Low input; unbiased information	Might not suitable for structured RNA; unable to tell m^6^Am and m^1^A
m^6^ACE-seq	Single base	5 μg mRNA	Transcriptomic m^6^A peaks	High resolution	Rely on exonuclease efficiency
SLIM-seq	100 −200 nt	100 ng total RNA	Transcriptomic m^6^A peaks	Ultra-low input	Relative low resolution
m^6^A-SEAL-seq	100–200 nt	5 μg mRNA	Transcriptomic m^6^A peaks	Cost-effective	Rely on FTO oxidation; low resolution
Nanopore-seq	Single base	50–100 ng RNA	DRACH-enriched sites*	Direct RNA sequencing; no cDNA bias	High error rate; computational demand
GEMS	Single cell	Living cells	Transcriptomic m^6^A peaks	Real-time readout of living cells	Limited to YTH domain-bound m^6^A signals
